# Characterization of Spbhp-37, a Hemoglobin-Binding Protein of *Streptococcus pneumoniae*

**DOI:** 10.3389/fcimb.2016.00047

**Published:** 2016-05-04

**Authors:** María E. Romero-Espejel, Mario A. Rodríguez, Bibiana Chávez-Munguía, Emmanuel Ríos-Castro, José de Jesús Olivares-Trejo

**Affiliations:** ^1^Departamento de Infectómica y Patogénesis Molecular, Centro de Investigación y de Estudios Avanzados del IPNMéxico, México; ^2^Unidad de Genómica, Proteómica y Metabolómica. LaNSE-CINVESTAV, Centro de Investigación y de Estudios Avanzados del IPNMéxico, México; ^3^Laboratorio de Bacteriología y Nanomedicina, Posgrado en Ciencias Genómicas, Universidad Autónoma de la Ciudad de MéxicoMéxico, México

**Keywords:** *Streptococcus pneumoniae*, haem, iron, iron starvation, haem-binding protein

## Abstract

*Streptococcus pneumoniae* is a Gram-positive microorganism that is the cause of bacterial pneumonia, sinusitis and otitis media. This human pathogen also can cause invasive diseases such as meningitis, bacteremia and septicemia. Hemoglobin (Hb) and haem can support the growth and viability of *S. pneumoniae* as sole iron sources. Unfortunately, the acquisition mechanism of Hb and haem in this bacterium has been poorly studied. Previously we identified two proteins of 37 and 22 kDa as putative Hb- and haem-binding proteins (Spbhp-37 and Spbhp-22, respectively). The sequence of Spbhp-37 protein was database annotated as lipoprotein without any function or localization. Here it was immunolocalized in the surface cell by transmission electron microscopy using specific antibodies produced against the recombinant protein. The expression of Spbhp-37 was increased when bacteria were grown in media culture supplied with Hb. In addition, the affinity of Sphbp-37 for Hb was determined. Thus, in this work we are presenting new findings that attempt to explain the mechanism involved in iron acquisition of this pathogen. In the future these results could help to develop new therapy targets in order to avoid the secondary effects caused by the traditional therapies.

## Introduction

*Streptococcus pneumoniae* is the most important cause of bacterial pneumonia and moreover this pathogen can cause infections as septicemia, bacteremia, and meningitis (Yaro et al., 2006; Thornton et al., 2010). This bacterium causes considerable human morbidity and mortality throughout the world, especially among children, the elderly and immunocompromised individuals (Gray et al., 1979; Austrian, 1989; Musher, 1992; Butler and Schuchat, 1999). However, the mechanisms for pneumococcal disease are not fully understood. There is a necessity for the discovering of novel therapeutic strategies focused on bacterial iron acquisition systems, because many bacteria pathogens require iron as an essential nutrient to infect the human (Klebba et al., 1982; Ratledge and Dover, 2000; Simpson et al., 2000; Crosa and Walsh, 2002; Andrews et al., 2003). Due to that the iron is required in several cellular processes, most bacteria have developed strategies for iron scavenging from host proteins (Wooldridge and Williams, 1993; Raymond et al., 2003; Ge and Sun, 2012; Andrews et al., 2013). One of the best studied bacterial iron acquisition systems is based on siderophores, which are secreted from the bacterial cell to scavenge free iron (Wooldridge and Williams, 1993; Guerinot, 1994; Wandersman and Delepelaire, 2004). Even though many pathogens secrete siderophores for iron acquisition during infection (Wandersman and Stojiljkovic, 2000; Genco and Dixon, 2001; Wandersman and Delepelaire, 2004), there are not biochemical or genetic evidences that *S. pneumoniae* produces siderophores (Tai et al., 1993; Brown et al., 2001; Romero-Espejel et al., 2013). As a result of the powerful reactivity of haem, it is generally sequestered within human cells by hemoproteins such as hemoglobin (Hb; Wandersman and Stojiljkovic, 2000; Wandersman and Delepelaire, 2004). In accordance, many bacteria have developed systems involved in iron acquisition from host hemoproteins (Tai et al., 1993; Brown et al., 2001; Genco and Dixon, 2001; Romero-Espejel et al., 2013). There are several studies on bacterial haem acquisition systems based mostly on Gram-negative bacteria (Stojiljkovic et al., 1996; Lewis et al., 1998; Wandersman and Stojiljkovic, 2000; Genco and Dixon, 2001; Olczak et al., 2001). Comparatively, less is known about how Gram-positive pathogens utilize host hemoproteins as an iron source. Recently, some surface proteins of *Streptococcus pyogenes* have been shown that bind haem (Shr and Shp, and haem-specific ATP-binding cassette transporter HtsABC). Shp has been shown to rapidly transfer its haem to the HtsA lipoprotein of HtsABC (Lei et al., 2002, 2003; Bates et al., 2003). In addition, it has been proposed that Shr is a source of haem for Shp and that the Shr-to-Shp haem transfer is a step of the haem acquisition process in *S. pyogenes* (Zhu et al., 2008).

*Staphylococcus aureus* acquires iron from haem by the Isd (iron-regulated surface determinant) system, which is formed by cell wall-anchored surface proteins (IsdA, IsdB, IsdC, and IsdH), a membrane transporter (composed by IsdD, IsdE, and IsdF), a transpeptidase (SrtB), and cytoplasmic haem-degrading monooxygenases (IsdG and IsdI) (Mazmanian et al., 2000, 2002, 2003; Skaar and Schneewind, 2004; Wu et al., 2005). Unfortunately, the mechanism of Hb and haem uptake in *S. pneumoniae* has been poorly studied. This pathogenic bacterium can grow using Hb or haem as a sole iron source. Hb acquisition is vital to microbial survival (Tai et al., 1993; Brown et al., 2001; Romero-Espejel et al., 2013). Previously, we detected two potential *S. pneumoniae* Hb- and haem-binding proteins (Spbhp) of 22 and 37 kDa, termed by us as Spbhp-22 and Spbhp-37. The Spbhp-37 protein had homology with a lipoprotein (Bierne et al., 2002; Romero-Espejel et al., 2013). Interestingly, several proteins required for virulence in Gram-positive bacteria are lipoproteins; for instance, FhuD which is an iron-siderophore transporter (Schneider and Hantke, 1993). Therefore, the aim of this work was to confirm the role of Spbhp-37 as Hb-binding protein and to determinate the affinity of Sphbp-37 for Hb.

## Materials and methods

### *S. pneumoniae* growth conditions

*S. pneumoniae* strain R6 was grown under microaerophilic conditions in 5% CO_2_ for 24 h at 37°C on agar plates supplemented with 5% sheep blood. The cellular cultures were then inoculated in plates containing Todd-Hewitt Broth (THB), supplemented with 0.5% yeast extract (THB-Y) and incubated for 16 h at 37°C with 5% CO_2_. For testing alternative iron sources the bacteria were cultivated in well culture plates containing medium THB, supplemented with 0.5% yeast extract (THB-Y) and 700 μm of 2,2′dipyridyl (a chelating agent) was added to eliminate free iron from the culture medium. Then, incubation was followed for 16 h at 37°C with 5% CO_2_. The cellular growth was adjusted to 0.1 (OD_600_) by spectrophotometry. After 3 h under iron starvation, the culture medium was supplemented with 2 μM human Hb.

### Cloning and expression of Spbhp-37 recombinant protein

The coding region of the *Spbhp-37* gene, excluding the signal peptide, was amplified by PCR from *S. pneumoniae* genomic DNA. For its directional cloning we used as sense primer an oligonucleotide containing the BamHI recognition site (5′-GGGGGGGATCCATGAACAAGAAACAATGGCTAGGTC-3′), and as anti-sense primer an oligonucleotide that included the SalI recognition site (5′-GGGGGGTCGACTTATTTT TCAGGAACTTTTACGCTTCCATC-3′). Then, amplicon was cloned in frame with the glutathione-S-transferase (GST), tag of the pGEX-6P-1 construction vector (GE Healthcare) using the BamHI and SalI restriction sites. The nucleotide sequence was corroborated by automated DNA sequencer.

For expression, *Escherichia coli* (strain BL21) competent cells were transformed with the pGEX-6P-1 empty vector, used as a negative control, or with the construction containing the *Spbhp-37* gene (pGEX-spbhp-37). Induction of recombinant proteins (GST and Spbhp-37-GST) was induced with 1 mM isopropyl-β-d-thiogalactopyranoside (IPTG) for 3 h at 37°C.

### Purification of the Spbhp-37 recombinant protein

Inclusion bodies (IB), where the recombinant protein was accumulated, were purified as described (Vallejo et al., 2002). Briefly, after protein induction, cultures were centrifuged at 1500 g for 40 min and bottom was suspended in 50 ml of buffer A (100 mM Tris-HCl pH 8.0, 10 mM EDTA, 100 mM NaCl) in the presence of 1 mM PMSF. Cells were sonicated for 30 s (100 W) and 50 s off time for a total sonication time (including the off time) of 10 min. Then, an equal volume of buffer A having 8 M urea and 1 mM PMSF was added, stirred for 1 h at 4°C and centrifuged at 10000 g for 30 min. Pellet was resuspended in 500 ml of buffer B (100 mM Tris-HCl pH 8.0, 1 mM EDTA, 1 M NaCl) and centrifuged at 10000 g for 30 min. Pellet was resuspended in 500 ml of water, centrifuged again, and frozen at −70°C. After that, pellet was resuspended in buffer C (2 M urea, 20 mM Tris-HCl pH 8.0, 0.5 M NaCl, and 2% Triton X-100), centrifuged for 15 min at 10000 g, washed with the same volume of buffer B and centrifuged again. Then, the wet pellet of IB (2.1 g) was dissolved in 20 ml of solubilization buffer (8 M urea, Tris-HCl pH 8.0, 0.5 M NaCl and 1 mM 2-mercaptoethanol) and stirred for 2 h at room temperature. Sample was centrifuged at 12000 g for 30 min at 4°C and supernatant was extensively dialyzed against a freshly prepared solution containing 20 mM Tris-HCl pH 8.0 and 4 mM urea. Finally, dialyzed samples were filter through a 0.45 μm membrane and stored in aliquots at −70°C. Recombinant protein was then purified by affinity chromatography using Glutathione–agarose beads (GE Healthcare) following the manufacturer's recommendations. Induction of the recombinant protein and its purification were examined by SDS-PAGE and western blotting assays using antibodies against GST. Cleavage of the GST tag was achieved using Pre-Scission protease following the manufacturer's recommendations.

### Production of specific antibodies

Recombinant protein was used as antigen to produce specific antibodies against Spbhp-37. Thus, recombinant protein was mixed with a volume of TiterMax Gold Adjuvant (Sigma 145380-33-2). Then, a New Zealand white rabbit was injected with 150 μg of protein in 1 ml of suspension. For immunization, protein suspension was divided in four doses of 250 μl each, which were injected into two subcutaneous and two intramuscular sites. Immunization was performed three times for periods of 15 days. The study was conducted in accordance with Good Laboratory Practices (GLP) and Use of Laboratory Animals (NOM-062-ZOO-1999). The study protocol was approved by the Institutional Animal Care and Use Committee (IACUC)-Cinvestav. Thereafter, antiserum was obtained and tested using total *S. pneumoniae* extracts or Spbhp-37 purified protein.

### Western immunoblotting

Protein samples from different fractions during purification of the Spbhp-37 recombinant protein or total extracts of *S. pneumoniae*, isolated as described (Romero-Espejel et al., 2013), were loaded onto 12% SDS-PAGE and transferred to nitrocellulose membranes. Membranes were soaked for 1 h with 5% non-fat milk in PBS in order to saturate all remaining active binding sites, and then they were incubated with anti-GST (glutathione transferase; 1:10000) or anti-Spbhp-37 (1:10000) antibodies. After that, membranes were incubated with anti-rabbit IgGs secondary antibodies conjugated to horseradish peroxidase (Invitrogen 65–6120; 1:10000) and the antibodies recognition was revealed by chemiluminescence (Millipore).

### Immunoelectron microscopy

Bacteria grown in THB-Y or in the presence of Hb as only iron source were fixed in 4% paraformaldehyde and 0.5% glutaraldehyde in PBS for 1 h at room temperature. Samples were embedded in the acrylic resin (LR White) and polymerized under UV at 4°C overnight. Thin sections (i.e., 60 nm) were obtained and mounted on Formvar-covered nickel grids. Later, sections were incubated in PBS with 10% fetal bovine serum before incubation with the anti-Spbhp-37 antibodies diluted (1:100) in 5% fetal bovine serum. Then, samples were incubated with anti-rabbit IgGs secondary antibodies conjugated to 20 nm colloidal gold spheres (Ted Pella Inc; 1:100). Finally, sections were contrasted with aqueous solutions of uranyl acetate and lead citrate before being examined in a Jeol JEM-1011 transmission electron microscope.

### *S. pneumoniae* growth in the presence of Hb and anti Spbhp-37-GST antibodies

Cells of *S. pneumoniae* previously cultivated and inoculated in THB (supplemented with 0.5% of yeast extract). When it was necessary to test an iron supply alternative, a chelating agent 700 μm 2,2′-dipyridyl (Sigma D216305-25G) was used in order to eliminate iron from the medium of culture. Thereafter, the bacteria were incubated for 16 h at 37°C with an atmosphere regulated at 5% CO_2_. In order to synchronize the cellular growth (OD_600_) was adjusted to 0.1. Thus, the cellular growth was monitored each hour. After 3 h under iron starvation the medium of culture was supplemented with: (a) only 2.5 μM of Hb (Cat. H7379, Sigma®), (b) pre-immune serum (500 mg/ml) plus Hb or (c) anti-Spbhp-37 antibodies (500 mg/ml) plus Hb. After that, cellular growth was monitored each hour for 4 h, comparing under iron limiting condition vs. the condition when Hb was supplied as the sole iron source or when the anti-Spbhp-37 antibodies were added.

### Overlay assays

Total extracts of *S. pneumoniae* were separated by 12% SDS-PAGE and transferred to nitrocellulose membranes (Bio-Rad). Membranes were incubated for 1 h at 37°C with 0.5% non-fat milk in PBS and 0.05% Tween 20, pH 7.4 (PBST) to block unspecific sites and then overnight at 4°C with human Hb (2.5 μM). After that, membranes were incubated for 1 h at 37°C with anti-hemoglobin antibodies (Santa Cruz Biotechnology SC-21005) (1:10000), and lastly 1 h at 37°C with the horseradish peroxidase-conjugated secondary antibodies (Invitrogen 65-6120; 1:10000). Antibodies recognition was developed by chemiluminescence (Millipore). As a control, a membrane was incubated only with PBST before the incubation with anti-globin antibodies.

### Surface plasmon resonance (SPR)

All SPR experiments were performed using Biacore T200 optical biosensor (GE Healthcare Life Sciences, Little Chalfont, Buckinghamshire, UK). SPR measurements were carried out in HBS-EP running buffer 10X (10 mM HEPES, 3 mM EDTA, 150 mM NaCl, 0.05% v/v of Tween 20 and pH 7.4) at 25°C in a CM5 chip (coated with carboxylated dextran). For the immobilization scouting Hb was dissolved (30 μg/ml) in acetate buffer (pH 3.5, 4, 4.5, 5, and 5.5) and injected at the Biacore system at flow rate of 10 μl/min, with a contact time of 120 s and using NaOH 50 mM as a wash solution to regenerate the chip surface. Once pH was selected (pH 4), Hb was dissolved in a corresponding acetate buffer (30 μg/ml) and immobilized to the chip using amine coupling at an immobilization level of *RL* = 435 *RU* to reach a theorical *RU*max = 249.53. During the coupling, the chip surface was activated using 1:1 mixture of 100 mM Nethyl-N-(dimethylaminopropyl)-carbodiimide (EDC) and 100 mM N-hydroxysuccinimide (NHS; both dissolved in water), and after Hb injection, the residual activated carboxy methyl groups on the chip surface were blocked by 1 M ethanolamine, pH 8.5. For this study, flow cell 1 was blank immobilized (without protein) for using as a reference. To analyze interactions of Spbhp-37 with immobilized Hb, Spbhp-37 was dissolved in buffer HBS-EP and injected. The same buffer was used as the running buffer. The flow rate was maintained constant throughout the kinetics experiment (30 μl/min), contact time was settled for 120 s and dissociation time was kept at 300 s. Regeneration was carried out with NaCl 1 M for 30 s. Experiments were performed with various concentrations of Sphbp-37 from 100 to 1000 nM, monitoring the refractive index changes as a function of time under constant flow conditions. The relative amount of Spbhp-37 bound to the Hb was determined by measuring the net increase of refractive index over time compared with that of running buffer alone. This change was reported in response units (*RU*). The data analysis was done with Biacore T200 evaluation software version 1.0 and data was fit to 1:1 binding.

## Results

### Cloning and expression of Spbhp-37

**Figure 1:**
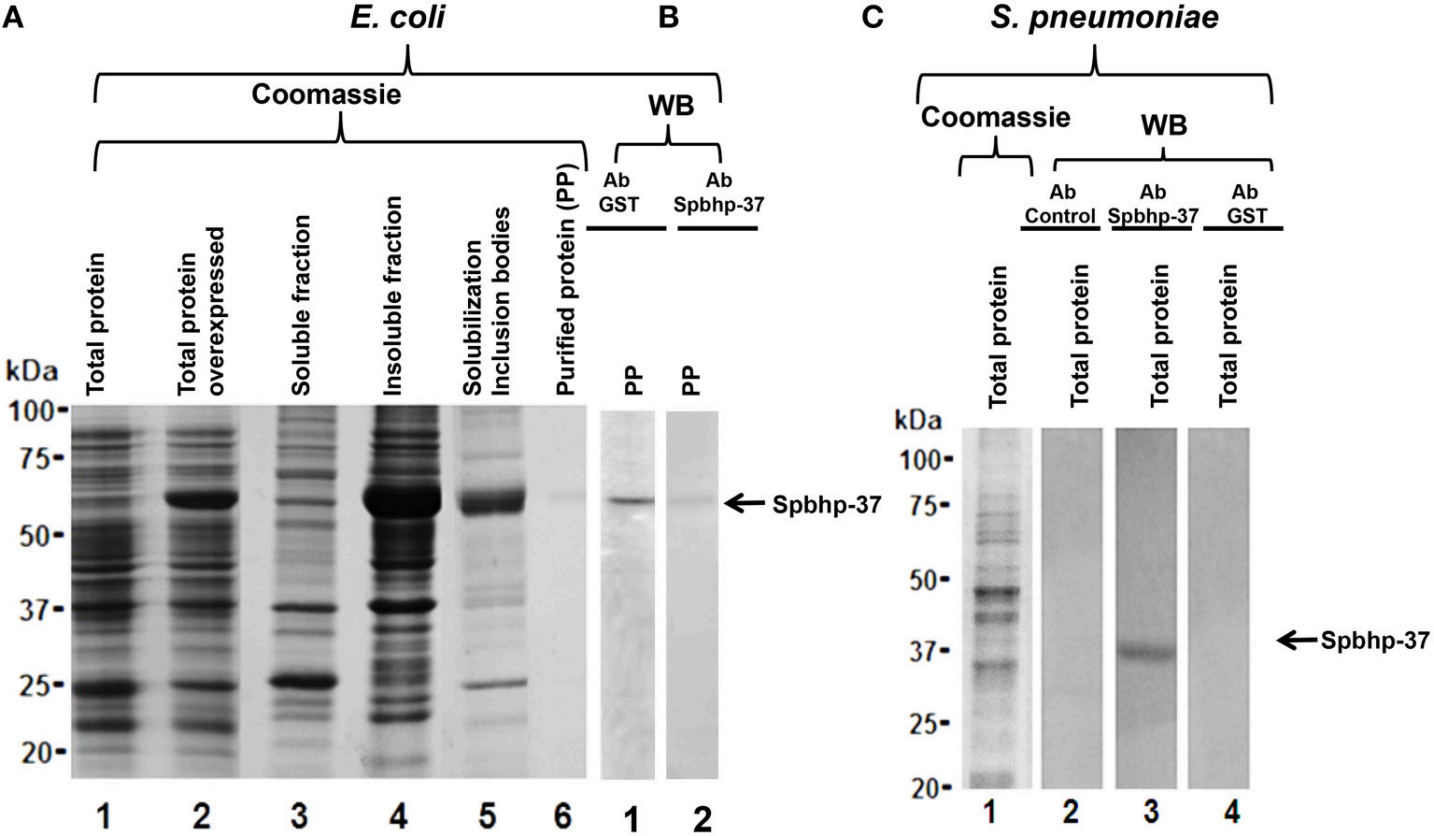
**Production of a Spbhp-37 recombinant protein and antibodies against it**. The Spbhp-37 encoding gene was cloned in the pGEX-6P-1 vector, the recombinant protein was expressed in *E. coli* bacteria (BL21 strain) and purified. Then, this protein was used as antigen to obtain specific antibodies against it. Finally, antibodies were utilized in western blotting assays on the recombinant protein and total extracts of *S. pneumoniae*. **(A)** Production and purification of the Spbhp-37 recombinant protein. Proteins were analyzed by 12% SDS-PAGE stained with coomassie blue. Lane 1, total proteins of non-induced bacteria; lane 2, total proteins of IPTG-induced bacteria; lane 3, soluble fraction of IPTG-induced bacteria; lane 4, insoluble fraction of IPTG-induced bacteria; lane 5, solubilization of inclusion bodies; lane 6, purified protein. **(B)** Western blotting on purified recombinant protein. Lane 1, western blotting using an antibody directed against the GST tag; lane 2, western blotting using an antibody directed against the recombinant protein (anti-Spbhp-37). **(C)** Proteins were analyzed by 12% SDS-PAGE stained with coomassie blue and western blotting on total proteins of *S. pneumoniae*. Lane 1, total extracts of *S. pneumoniae* were analyzed by 12% SDS-PAGE stained with coomassie blue; lane 2, western blotting using the pre-immune serum; lane 3, western blotting using anti-Spbhp-37 antibodies; lane 4, western blotting using anti-GST. Molecular weight markers are indicated on the left. Arrows indicate the antibodies recognition of recombinant protein Spbhp-37.

To investigate the participation of Spbhp-37 protein on iron acquisition, its encoding gene was cloned in the plasmid pGEX-6-P-1. The construction was termed pGEX-spbhp-37 and was used to transform BL21 strain; the expression of Spbhp-37 was induced with IPTG. The overexpression of Spbhp-37 protein was confirmed by SDS-PAGE stained with Coomassie blue, we observed a band of 63 kDa when bacteria were incubated with IPTG with respect to non-induced bacteria (Figure 1, lane 2). This molecular weight corresponds to the expected for the recombinant protein (26 kDa from GST and 37 kDa from Spbhp-37). Unfortunately, when soluble and insoluble fractions were separated, the Spbhp-37 recombinant protein was detected in the insoluble fraction (Figure 1, lane 4). Therefore, a protocol to solubilize the inclusion bodies (IB) was performed (see Materials and Methods Section) (Figure 1, lane 5) previous to purification of the protein by affinity chromatography (Figure 1, lane 6). To confirm the identity of the purified protein, we carried out western blot assays using anti-GST antibodies. Results showed that antibodies recognized the purified recombinant protein (Figure 1B, lane 1).

### Obtaining of anti Spbhp-37 antibodies

In order to produce anti Spbhp-37 antibodies, the recombinant protein was used as antigen to inoculate a New Zealand rabbit. As expected, the obtained antibodies recognized the recombinant protein in western blot assays (Figure 1B, lane 2). After that, antibodies were characterized by western blotting on total proteins of *S. pneumoniae*. In these assays, antibodies recognized a single band of 37 kDa, which corresponds to the molecular weight of Spbhp-37 (Figure 1C, lane 3). This band was not revealed when western blotting was performed with pre- immune serum or with anti-GST antibodies (Figure 1C, lanes 2, 4, respectively). These results allowed us to demonstrate the specificity of the antibodies raised against Spbhp-37.

### Spbhp-37 protein is increased two fold on the surface of *S. pneumoniae* when it is grown in the presence of Hb as only iron source

**Figure 2:**
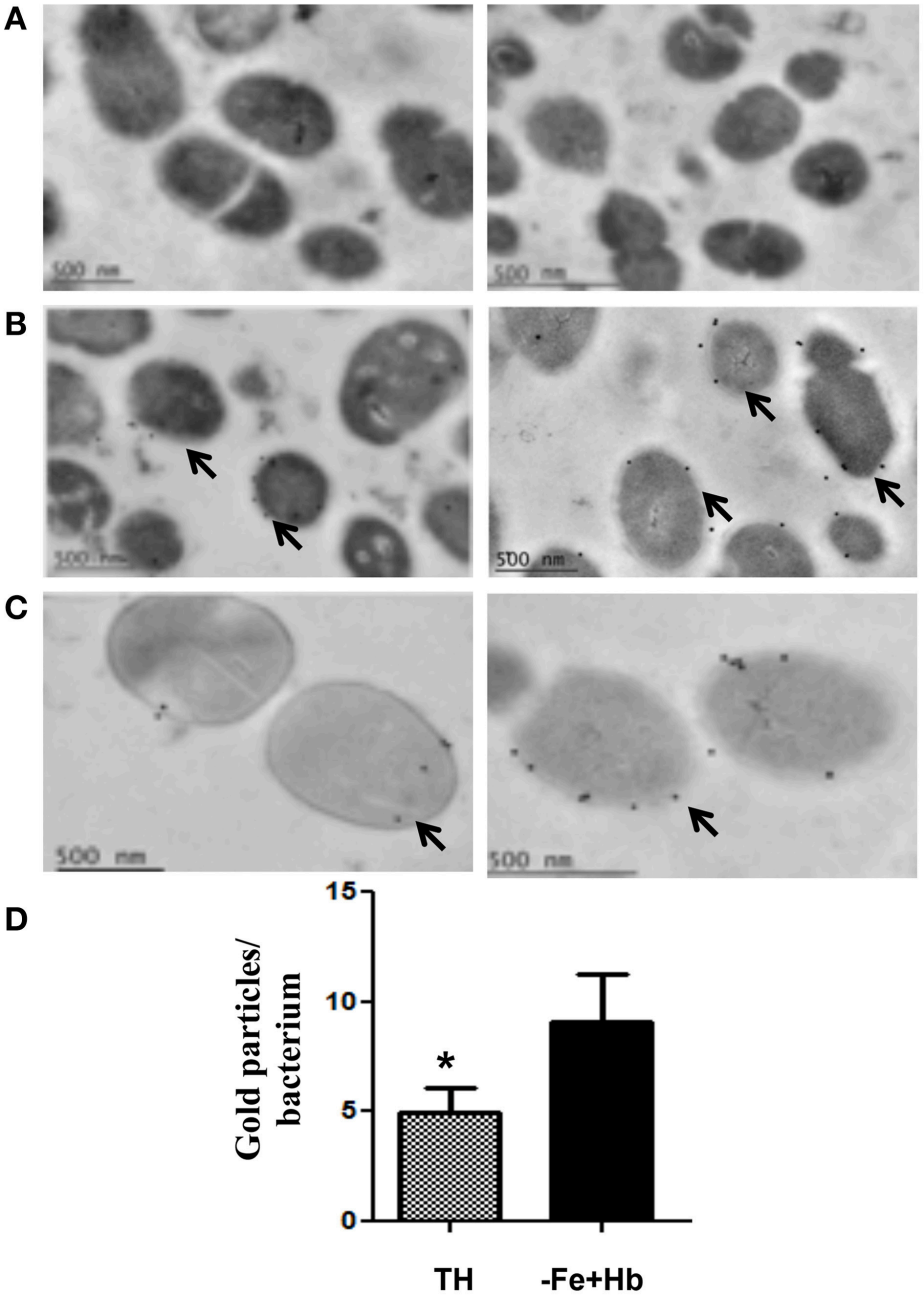
**Immunoelectron microscopy of Spbhp-37 protein**. *S. pneumoniae* cellular cultures were incubated in normal Todd-Hewitt Broth or in medium with Hb as only iron source. Then, localization of Spbhp-37 was analyzed by immunoelectron microscopy. **(A)** Negative control. Bacteria incubated only with the gold-labeled secondary antibodies. **(B)** Spbhp-37 in normal medium. **(C)** Spbhp-37 in medium with Hb as only iron source. Arrowheads indicate the location of Spbhp-37 protein in *S. pneumoniae*. In right is shown the magnification of cell bacteria to compare expression in both growth conditions. **(D)** Quantitative analysis of Spbhp-37 expression. Gold particles on bacteria growth under normal conditions (TH) and in medium with Hb as only iron source (-Fe+Hb) were counted *n* = 25. Data represent mean ± *SD* of three independent experiments. Asterisk indicates a significant difference (*p* < 0.05).

To investigate the location of Spbhp-37 in *S. pneumoniae* bacteria we performed immunoelectronic microscopy assays using antibodies against the recombinant protein. Our results showed the presence of Spbhp-37 protein on bacteria surface (Figure 2B). Signal was specific for Spbhp-37 because not signal was detected in a control incubated only with the gold-labeled secondary antibodies (Figure 2A). Then, to analyze the effect of Hb on the expression of Spbhp-37, we cultivated *S. pneumoniae* in THB in the presence of Hb as the sole iron source. In this condition we observed that the occurrence of Spbhp-37 on bacteria surface was more abundant than when cells were grown in normal medium (Figure 2C). To obtain a quantitative value, the positive signals were counted in each condition. Results revealed an increase of about two fold of Spbhp-37 in bacteria grown with Hb (Figure 2D). This result was corroborated by western blotting (data no showed). These results suggest that the presence of Hb increases two fold the abundance of Spbhp-37.

### Anti Spbhp-37-GST antibodies limited the cellular growth when Hb was supplied as the sole iron source

**Figure 3:**
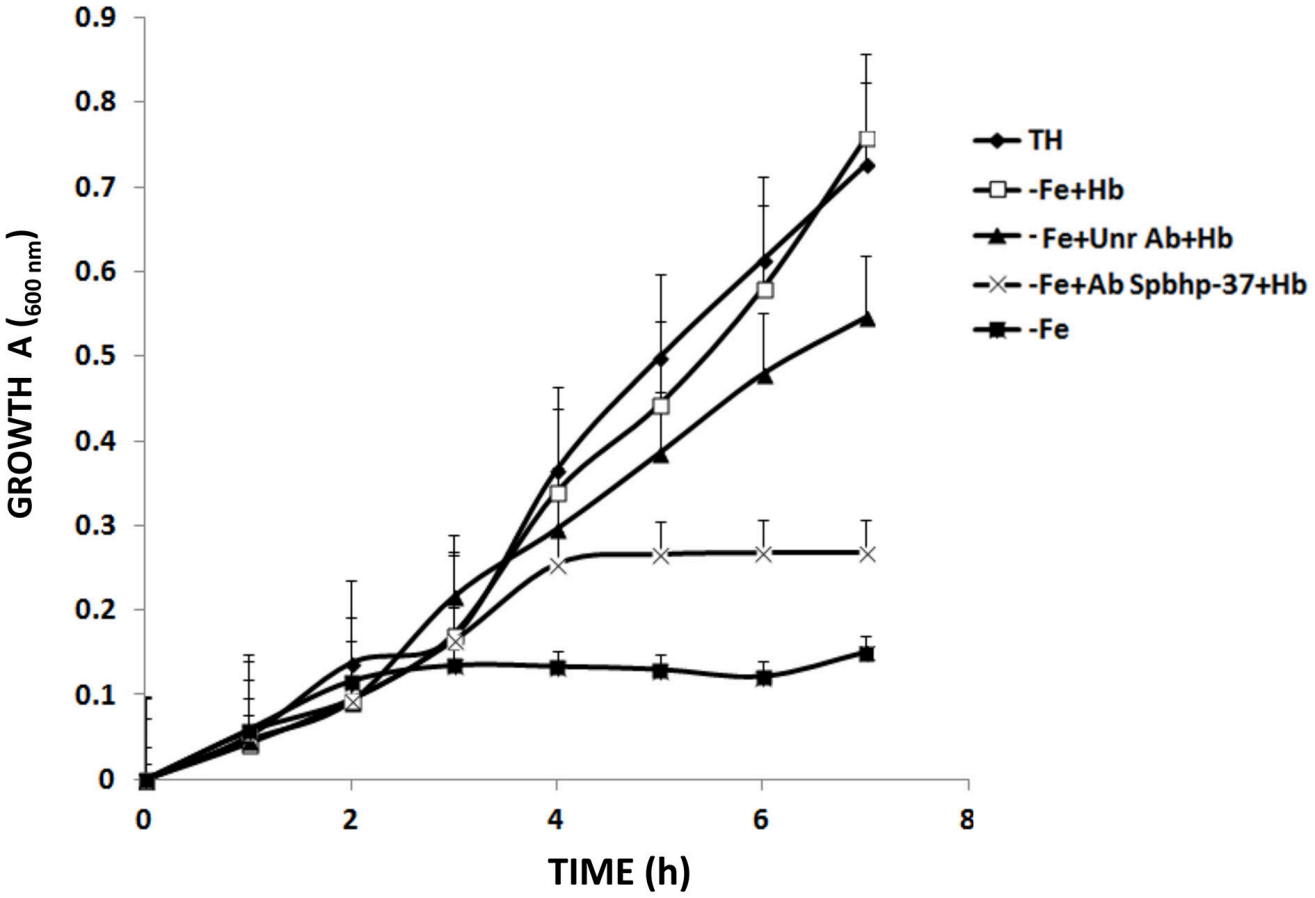
**Anti Spbhp-37-GST antibodies block the cellular growth of ***S. pneumoniae*****. *S. pneumoniae* strain R6 was cultivated in Todd-Hewitt Broth under conditions of: iron sufficiency (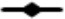); without iron (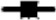); without iron and supplemented with Hb (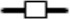); without iron and supplemented with Hb in the presence of anti-Spbhp-37 antibodies (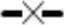); and without iron and supplemented with Hb in the presence of pre-immune serum (used as unrelated antibodies) (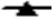). The cellular growth was monitored each hour for a period of 7 h by spectrophotometry (600 nm). Data represent mean ± *SD* of three independent experiments by triplicate. The cellular growth from bacteria cultivated in the medium without iron and supplemented with Hb in the presence of anti-Spbhp-37 antibodies was significantly higher compared to the cellular growth from bacteria cultivated in the medium without iron and supplemented with Hb in the presence of pre-immune serum (used as unrelated antibodies; *p* < 0.05, one-way ANOVA).

To explore whether Spbhp-37 protein is related to utilization of Hb in the cellular growth of *S. pneumoniae*, we designed an experiment in which the anti-Spbhp-37 antibodies were supplied to block the bacteria growth under free iron limiting conditions, but using Hb as sole iron source. When *S. pneumoniae* was cultivated in THB under iron starvation, the cellular growth was limited, but when this media was supplemented with Hb as the sole iron source, the cellular growth was restored (Figure 3). Interestingly, the cellular growth was blocked when anti-Spbhp-37 antibodies were added to cellular cultures with Hb as the sole iron source (Figure 3). In addition, pre-immune serum had a minimal effect on the cellular growth in media supplemented with Hb (Figure 3). Results clearly showed the cellular growth under iron limiting conditions was impaired.

### Anti-Spbhp-37 antibodies block the interaction between Spbhp-37 protein and Hb

**Figure 4:**
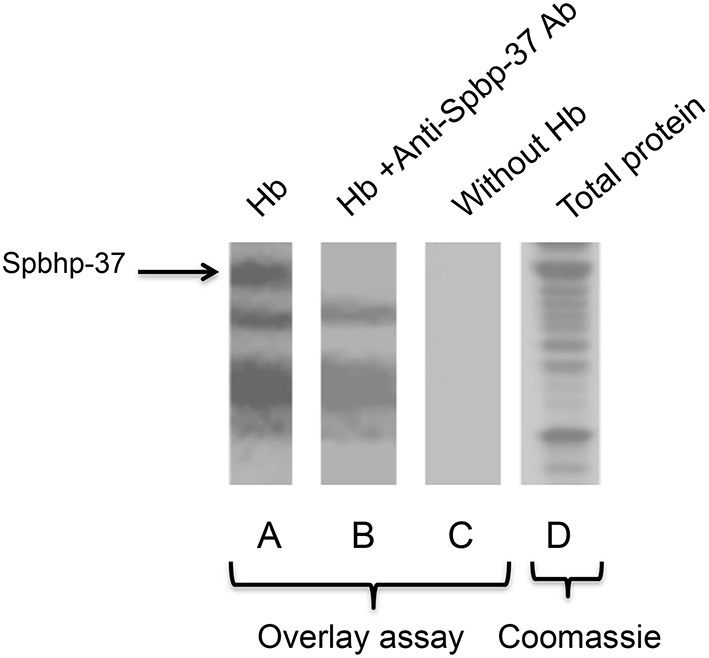
**Hb binds to Spbhp-37 protein in overlay experiments**. Total proteins of *S. pneumoniae* were loaded onto 12% SDS-PAGE, transferred to nitrocellulose membrane and overlay assays were performed. **(A)** Overlay. Nitrocellulose membranes were incubated with Hb, with anti-hemoglobin antibodies, and peroxidase-conjugated secondary antibodies. **(B)** As in A, but the overlay assay was incubated first with anti-Spbhp-37 antibodies. **(C)** Negative control. Nitrocellulose membranes were incubated only with primary and secondary antibodies (omitting the incubation with Hb). **(D)** Coomassie blue staining. Total proteins of *S. pneumoniae* were loaded onto 12% SDS-PAGE. Arrow indicates the Spbhp-37 protein.

Inhibition of cellular growth with anti-Spbhp-37 notwithstanding the presence of Hb supports the hypothesis that Spbhp-37 is a receptor for Hb and that antibodies block the interaction between both proteins. To corroborate these assumptions, the interaction between Spbhp-37 protein and Hb as well the blockage of this interaction by anti Spbhp-37 antibodies were investigated by overlay assays. Thus, total proteins of *S. pneumoniae* (Figure 4D) were separated in SDS-PAGE and transferred to nitrocellulose membranes. Then, membranes were incubated with Hb and interaction was revealed with anti-Hb antibodies. At least five proteins, including a 37 kDa band Spbhp-37 protein (located in the top of gel), were recognized by Hb and anti-Hb antibodies when total proteins were used (Figure 4A). Interestingly, the detection of the protein was not observed when anti-Spbhp-37 antibodies were added in the overlay assays (Figure 4B). In addition, not bands were detected when incubation of Hb was omitted in the overlay experiments (used as a negative control) (Figure 4C). Results confirmed that Spbhp-37 binds to Hb and that antibodies block the interaction between both proteins.

### Spbhp-37 showed high affinity by Hb

**Figure 5:**
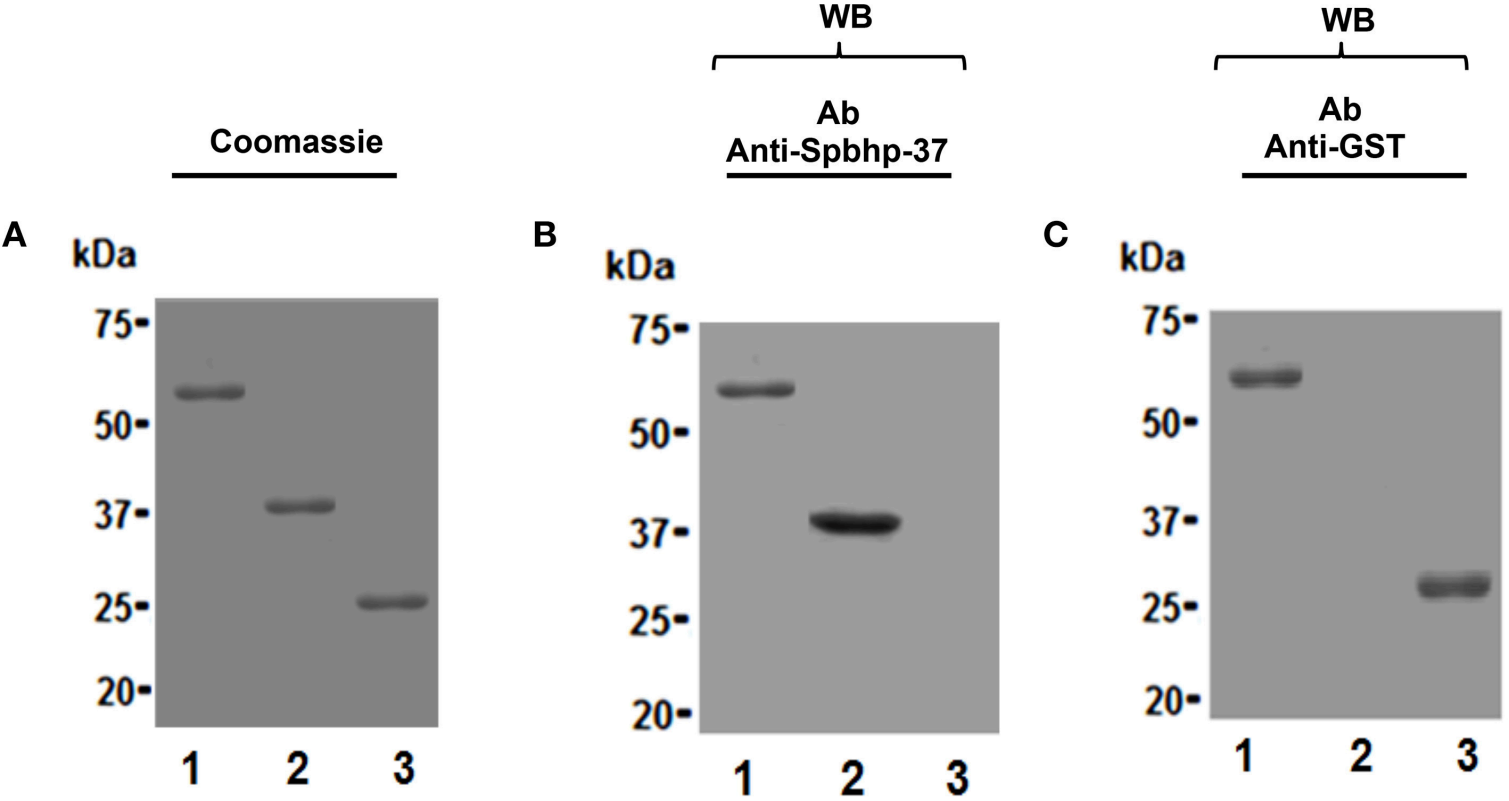
**Purification of the Spbhp-37 recombinant protein without the GST tag**. Spbhp-37-GST recombinant protein was purified by chromatography affinity and the cleavage of GTS tag was performed by the PreScission protease. Then, protein samples were analyzed by 12% SDS-PAGE and western blotting using anti-Spbhp-37 antibodies was tested. **(A)** Coomassie blue staining. **(B)** Western blotting with anti-Spbhp-37. **(C)** Western blotting with anti-GST. Lane 1, Spbhp-37-GST; lane 2, Spbhp-37 without GST; lane 3, GST. Molecular weight markers are indicated at the left side.

To determine the affinity of Spbhp-37 for Hb, the GST tag of the Spbhp-37 recombinant protein was eliminated by digestion with the Pre-Scission protease and its affinity to Hb was analyzed by surface plasmon resonance (SPR). First, to confirm the cleavage of the GST tag, protein samples were analyzed by SDS-PAGE and western blotting. After SDS-PAGE and coomassie blue staining we observed the purified Spbhp-37-GST protein (Figure 5A, lane 1), the Spbhp-37 protein without GST (Figure 5A, lane 2), and the releasing of the GST tag (Figure 5A, lane 3). Identity of Spbhp-37 protein was corroborated by western blotting assays using the anti Spbhp-37 antibodies. These antibodies recognized the Spbhp-37-GST protein (Figure 5B, lane 1) and the Spbhp-37 protein without the GST tag (Figure 5B, lane 2), but GST was not revealed (Figure 5B, lane 3). As a control, western blotting was performed using an anti-GST antibody, where only Spbhp-37-GST protein (Figure 5C, lane 1), and GST (Figure 5C, lane 3) were revealed.

**Figure 6:**
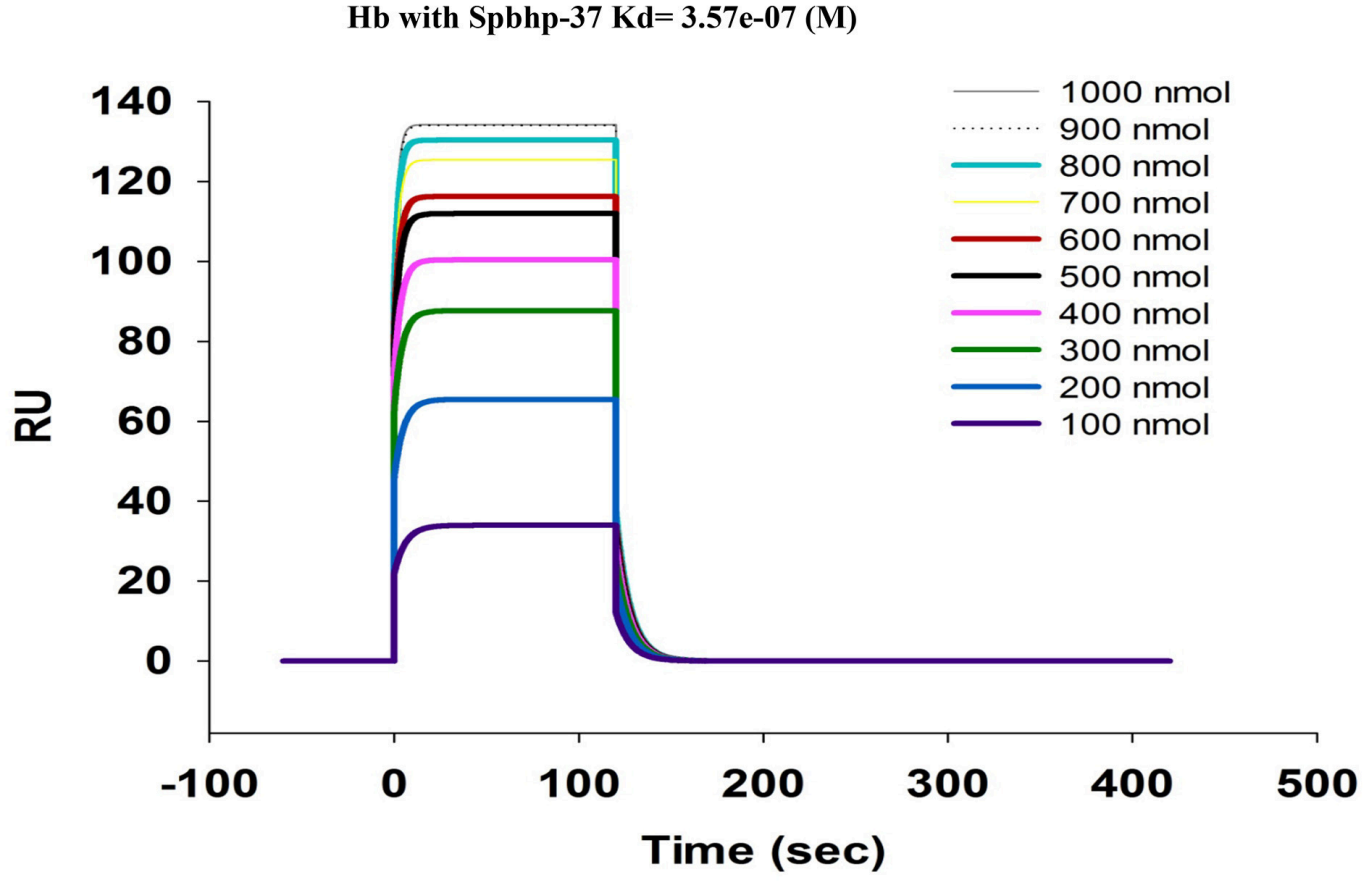
**Quantitative analysis of hemoglobin-Sphbp-37 interaction using Biacore assay (Surface Plasmon Resonance)**. Human hemoglobin was immobilized on sensor chips CM5 (Biacore) and binding curves for Spbhp-37 were expressed in resonance units (*RU*) as a function of time. The kinetic parameters and concentrations of the analysis are indicated.

To obtain kinetic binding data by SPR, Hb was immobilized on the sensor chip and the binding of Spbhp-37 (without GST tag) at concentrations ranged from 100 to 1000 nm was tested. In these experiments we observed a dose-dependent binding of Spbhp-37 to Hb (Figure 6). Curve fitting of the sensograms enabled us to determine that Kd was of 3.57 e-7 M, showing a high affinity of Spbhp-37 protein for Hb.

## Discussion

*S. pneumoniae* is a human pathogen that uses Hb to cover its iron necessities. However, the iron acquisition mechanism has been poorly studied. Previously, we identified a lipoprotein of 37 kDa (Spbhp-37) as a *S. pneumoniae* membrane protein, which was purified by haem affinity chromatography and that could be involved in the Hb and haem acquisition. In the present work, by use of an antibody raised against Spbhp-37 recombinant protein we showed that the expression of native protein was increased on the surface of *S. pneumoniae* when Hb was supplemented as the sole iron source. These results clearly showed the character of receptor protein, because it was detected on the surface of the bacterium (Lei et al., 2002, 2003; Bates et al., 2003; Mazmanian et al., 2003). In addition, when these antibodies were used to analyze the bacterial growth in the presence of Hb, we noticed that they diminished about 50% the cell growth with respect to that obtained in media with Hb in the absence of this antibody or in the presence of pre-immune serum, indicating the importance of Spbhp-37 on uptake of Hb when was supplied as only iron source. This hypothesis was confirmed by overlay assays, because we found that Hb bound to Spbhp-37 protein and anti Spbhp-37 antibodies were capable to inhibit the interaction between Hb and Spbhp-37 protein. In fact, antibodies only blocked the Hb binding to one (Spbhp-37) of the all bands previously identified in *S. pneumoniae* total proteins (Romero-Espejel et al., 2013), which correspond to 37 kDa size, this observation clearly showed the specificity of the antibodies produced. On the other hand, the low Kd determined by SPR for the binding of Hb and Spbhp-37 protein (3.57 e^−7^ M) is similar to that reported for the TonB-dependent haem receptor (HasR) of *Serratia marcescens*, the outer membrane haem receptor (HmuR) of *Porphyromonas gingivalis*, and haem receptor (HasA) of *S. marcescens* (Ghigo et al., 1997; Olczak et al., 2001; Deniau et al., 2003), indicating that Spbhp-37 protein binds Hb with high affinity and suggesting that this protein is necessary in the mechanisms of iron acquisition to scavenge iron. Possibly other proteins are involved in this mechanism, for example those reported previously by our group, which include a stress general protein of 22 kDa, a maltose-binding protein of 45 kDa, and a glutamine synthetase type I 50 kDa (Romero-Espejel et al., 2013). These proteins could help to introduce and store the iron source to cytoplasm as it has been described for other Gram-positive bacteria (Mazmanian et al., 2002, 2003; Skaar et al., 2004; Skaar and Schneewind, 2004; Wu et al., 2005).

Our overall results attempt to explain the iron acquisition mechanism by *S. pneumoniae* when Hb is available. We showed that Spbhp-37 is a surface protein involved in Hb uptake, an essential mechanism of this pathogen to establish an infection process in human, because this bacterium uses Hb and haem as iron sources.

## Author contributions

ME conceived and carried out most of the experiments, analyzed data, and drafted the manuscript; BC was responsible for transmission electronic microscopy experiments; ER performed the surface plasmon resonance assays; MR and JO designed the study, analyzed data, and drafted the manuscript. All authors read and approved the final manuscript.

## Funding

This work was supported by Consejo Nacional de Ciencia y Tecnología (CONACyT) [grant numbers: SALUD-2012-01-181641 and 222180].

### Conflict of interest statement

The authors declare that the research was conducted in the absence of any commercial or financial relationships that could be construed as a potential conflict of interest.

## References

[B1] Andrews S. Norton I. Salunkhe A. S. Goodluck H. Aly W. S. Mourad-Agha H.. (2013). Control of iron metabolism in bacteria. Met. Ions Life Sci. 12, 203–239. 10.1007/978-94-007-5561-1_7 23595674

[B2] Andrews S. Robinsón A. K. Rodríguez-Quiñonez F. (2003). Bacterial iron homeostasis. FEMS Microbiol. Rev. 27, 215–237. 10.1016/S0168-6445(03)00055-X 12829269

[B3] Austrian R. (1989). Pneumococcal polysaccharide vaccines. Rev. Infect. Dis. 11, 598–602. 10.1093/clinids/11.Supplement_3.S598 2669103

[B4] Bates C. S. Montanez G. E. Woods C. R. Vincent R. M. Eichenbaum Z. (2003). Identification and characterization of a *Streptococcus pyogenes* operon involved in binding of hemoproteins and acquisition of iron. Infect. Immun. 71, 1042–1055. 10.1128/IAI.71.3.1042-1055.2003 12595414 PMC148835

[B5] Bierne H. Mazmanian S. K. Trost M. Pucciarelli M. G. Liu G. Dehoux P.. (2002). Inactivation of the srtA gene in *Listeria monocytogenes* inhibits anchoring of surface proteins and affects virulence. Mol. Microbiol. 43, 869–881 10.1046/j.1365-2958.2002.02798.x 11929538

[B6] Brown J. S. Gilliland S. M. Holden D. W. (2001). A *Streptococcus pneumoniae* pathogenicity island encoding an ABC transporter involved in iron uptake and virulence. Mol. Microbiol. 40, 572–585. 10.1046/j.1365-2958.2001.02414.x 11359564

[B7] Butler J. C. Schuchat A. (1999). Epidemiology of pneumococcal infections in the elderly. Drugs Aging 15, 11–19. 10.2165/00002512-199915001-00002 10690791

[B8] Crosa J. Walsh C. (2002). Genetics and assembly line enzymology of siderophore biosynthesis in bacteria. Microbiol. Mol. Biol. R. 66, 223–249. 10.1128/MMBR.66.2.223-249.2002 12040125 PMC120789

[B9] Deniau C. Gilli R. Izadi-Pruneyre N. Letoffe S. Delepierre M. Wandersman C.. (2003). Thermodynamics of heme binding to the HasA(SM) hemophore: effect of mutations at three key residues for heme uptake. Biochemistry 42, 10627–10633. 10.1021/bi030015k 12962486

[B10] Ge R. Sun X. (2012). Iron trafficking system in *Helicobacter pylori*. BioMetals 25, 247–258. 10.1007/s10534-011-9512-8 22127376

[B11] Genco C. A. Dixon D. W. (2001). Emerging strategies in microbial haem capture. Mol. Microbiol. 39, 1–11. 10.1046/j.1365-2958.2001.02231.x 11123683

[B12] Ghigo J. M. Letoffe S. Wandersman C. (1997). A new type of hemophore-dependent heme acquisition system of *Serratia marcescens* reconstituted in *Escherichia coli*. J. Bacteriol. 179, 3572–3579. 9171402 10.1128/jb.179.11.3572-3579.1997 PMC179150

[B13] Gray B. M. Converse J. Dillon H. (1979). Serotypes of *Streptococcus pneumoniae* causing disease. J. Infect. Dis. 140, 979–983. 10.1093/infdis/140.6.979 44310

[B14] Guerinot M. L. (1994). Microbial iron transport. Annu. Rev. Microbiol. 48, 743–772. 10.1146/annurev.mi.48.100194.003523 7826025

[B15] Klebba P. E. McIntosh M. A. Neilands J. B. (1982). Kinetics of biosynthesis of iron-regulated membrane proteins in *Escherichia coli*. J. Bacteriol. 149, 880–888. 6174499 10.1128/jb.149.3.880-888.1982 PMC216474

[B16] Lei B. Liu M. Voyich J. M. Prater C. I. Kala S. V. DeLeo F. R.. (2003). Identification and characterization of HtsA, a second heme-binding protein made by *Streptococcus pyogenes*. Infect. Immun. 71, 5962–5969. 10.1128/iai.71.10.5962-5969.2003 14500516 PMC201091

[B17] Lei B. Smoot L. M. Menning H. M. Voyich J. M. Kala S. V. Deleo F. R.. (2002). Identification and characterization of a novel heme-associated cell surface protein made by *Streptococcus pyogenes*. Infect. Immun. 70, 4494–4500. 10.1128/IAI.70.8.4494-4500.2002 12117961 PMC128137

[B18] Lewis L. A. Sung M. H. Gipson M. Hartman K. Dyer D. W. (1998). Transport of intact porphyrin by HpuAB, the hemoglobin-haptoglobin utilization system of *Neisseria meningitidis*. J. Bacteriol. 180, 6043–6047. 9811666 10.1128/jb.180.22.6043-6047.1998 PMC107682

[B19] Mazmanian S. K. Liu G. Jensen E. R. Lenoy E. Schneewind O. (2000). *Staphylococcus aureus* sortase mutants defective in the display of surface proteins and in the pathogenesis of animal infections. Proc. Natl. Acad. Sci. U.S.A. 97, 5510–5515. 10.1073/pnas.080520697 10805806 PMC25859

[B20] Mazmanian S. K. Skaar E. P. Gaspar A. H. Humayun M. Gornicki P. Jelenska J.. (2003). Passage of heme-iron across the envelope of *Staphylococcus aureus*. Science 299, 906–909. 10.1126/science.1081147 12574635

[B21] Mazmanian S. K. Ton-That H. Su K. Schneewind O. (2002). An iron-regulated sortase anchors a class of surface protein during *Staphylococcus aureus* pathogenesis. Proc. Natl. Acad. Sci. U.S.A. 99, 2293–2298. 10.1073/pnas.032523999 11830639 PMC122358

[B22] Musher D. M. (1992). Infections caused by *Streptococcus pneumoniae*: clinical spectrum, pathogenesis, immunity, and treatment. Clin. Infect. Dis. 14, 801–807. 10.1093/clinids/14.4.801 1576274

[B23] Olczak T. Dixon D. W. Genco C. A. (2001). Binding specificity of the *Porphyromonas gingivalis* heme and hemoglobin receptor HmuR, gingipain K, and gingipain R1 for heme, porphyrins, and metalloporphyrins. J. Bacteriol. 183, 5599–5608. 10.1128/JB.183.19.5599-5608.2001 11544222 PMC95451

[B24] Ratledge C. Dover L. (2000). Iron metabolism in pathogenic bacteria. Annu. Rev. Microbiol. 54, 881–941. 10.1146/annurev.micro.54.1.881 11018148

[B25] Raymond K. N. Dertz E. A. Kim S. S. (2003). Enterobactin: an archetype for microbial iron transport. Proc. Natl. Acad. Sci. U.S.A. 100, 3584–3588. 10.1073/pnas.0630018100 12655062 PMC152965

[B26] Romero-Espejel M. E. González-López M. A. Olivares-Trejo J. J. (2013). *Streptococcus pneumoniae* requires iron for its viability and expresses two membrane proteins that bind haemoglobin and haem. Metallomics 5, 384–389. 10.1039/c3mt20244e 23487307

[B27] Schneider R. Hantke K. (1993). Iron-hydroxamate uptake systems in *Bacillus subtilis*: identification of a lipoprotein as part of a binding protein dependent transport system. Mol. Microbiol. 8, 111–121. 10.1111/j.1365-2958.1993.tb01208.x 8388528

[B28] Simpson W. Olczak T. Genco C. A. (2000). Characterization and expression of HmuR, a TonB-dependent hemoglobin receptor of *Porphyromonas gingivalis*. J. Bacteriol. 182, 5737–5748. 10.1128/JB.182.20.5737-5748.2000 11004172 PMC94695

[B29] Skaar E. P. Gaspar A. H. Schneewind O. (2004). IsdG and IsdI, heme-degrading enzymes in the cytoplasm of *Staphylococcus aureus*. J. Biol. Chem. 279, 436–443. 10.1074/jbc.m307952200 14570922

[B30] Skaar E. P. Schneewind O. (2004). Iron-regulated surface determinants (Isd) of *Staphylococcus aureus*: stealing iron from heme. Microbes Infect. 6, 390–397. 10.1016/j.micinf.2003.12.008 15101396

[B31] Stojiljkovic I. Larson J. Hwa V. Anic S. So M. (1996). HmbR outer membrane receptors of pathogenic Neisseria spp.: iron-regulated, hemoglobin-binding proteins with a high level of primary structure conservation. J. Bacteriol. 178, 4670–4678. 8755899 10.1128/jb.178.15.4670-4678.1996 PMC178238

[B32] Tai S. S. Lee C. J. Winter R. E. (1993). Hemin utilization is related to virulence of *Streptococcus pneumoniae*. Infect. Immun. 61, 5401–5405. 8225615 10.1128/iai.61.12.5401-5405.1993 PMC281331

[B33] Thornton J. Durick-Eder K. Tuomanen E. (2010). Pneumococcal pathogenesis: “innate invasion” yet organ-specific damage. J. Mol. Med. 88, 103–107. 10.1007/s00109-009-0578-5 20162252 PMC2864529

[B34] Vallejo L. Brokelman M. Marten S. Trappe S. Cabrera J. Hoffmann A.. (2002). Renaturation and purification of bone morphogenetic protein-2 produced as inclusion bodies in high-cell-density cultures of recombinant *Escherichia coli*. J. Bacteriol. 94, 185–194. 10.1016/s0168-1656(01)00425-4 11796171

[B35] Wandersman C. Delepelaire P. (2004). Bacterial iron sources: from siderophores to haemophores. Annu. Rev. Microbiol. 58, 611–647. 10.1146/annurev.micro.58.030603.123811 15487950

[B36] Wandersman C. Stojiljkovic I. (2000). Bacterial heme sources: the role of heme, hemoprotein receptors and haemophores. Curr. Opin. Microbiol. 3, 215–220. 10.1016/S1369-5274(00)00078-3 10744995

[B37] Wooldridge K. G. Williams P. H. (1993). Iron uptake mechanisms of pathogenic bacteria. FEMS Microbiol. Rev. 12, 325–348 10.1111/j.1574-6976.1993.tb00026.x 8268005

[B38] Wu R. Skaar E. P. Zhang R. Joachimiak G. Gornicki P. Schneewind O.. (2005). Staphylococcus aureus IsdG and IsdI, heme-degrading enzymes with structural similarity to monooxygenases. J. Biol. Chem. 280, 2840–2846. 10.1074/jbc.M409526200 15520015 PMC2792019

[B39] Yaro S. Lourd M. Traoré Y. Njanpop-Lafourcade B. M. Sawadogo A. Sangare L.. (2006). Epidemiological and molecular characteristics of a highly lethal pneumococcal meningitis epidemic in Burkina Faso. Clin. Infect. Dis. 43, 693–700. 10.1086/506940 16912941

[B40] Zhu H. Liu M. Lei B. (2008). The surface protein Shr of *Streptococcus pyogenes* binds heme and transfers it to the streptococcal heme-binding protein Shp. BMC Microbiol. 8:15. 10.1186/1471-2180-8-15 18215300 PMC2266757

